# The Relationship Between Self-Compassion and Resilience in the General Population: Protocol for a Systematic Review and Meta-Analysis

**DOI:** 10.2196/60154

**Published:** 2024-12-05

**Authors:** Xinyi Li, Melina Aikaterini Malli, Theodore D Cosco, Guangyu Zhou

**Affiliations:** 1 School of Psychological and Cognitive Sciences Beijing Key Laboratory of Behavior and Mental Health Peking University Beijing China; 2 Oxford Institute of Population Ageing University of Oxford Oxford United Kingdom; 3 Department of Gerontology Simon Fraser University Vancouver, BC Canada

**Keywords:** self-compassion, resilience, resiliency, adversity, compassion, coping, coping styles, health status, meta-analysis, meta-regression

## Abstract

**Background:**

Resilience can protect individuals from the negative impact of adversity, facilitating a swift recovery. The exploration of protective factors contributing to resilience has been a central focus of research. Self-compassion, a positive psychological construct that involves treating oneself with kindness, holds the potential to bolster resilience. Although several studies have indicated an association between self-compassion and resilience, there is a lack of systematic reviews and meta-analyses examining this relationship and the potential moderators and mechanisms.

**Objective:**

This study aimed to systematically review the literature on the relationship between self-compassion and resilience in the general population, perform a meta-analysis to quantify the effect size of their association, and explore potential moderators (eg, age, gender, culture, and health status) and mediators.

**Methods:**

We will search the Web of Science, PsycINFO, MEDLINE, Scopus, CINAHL, and CNKI databases for peer-reviewed studies (including observational and experimental studies) that examined the relationship between self-compassion and resilience, with no language restrictions. There are no restrictions regarding participants’ age, gender, culture, or health status. Qualitative studies, conference abstracts, review articles, case reports, and editorials will be excluded. Two reviewers (XL and JH) will independently screen the literature, extract data, and assess the quality of the eligible studies. If possible, the pooled effect size between self-compassion and resilience will be meta-analyzed using a random-effect model. Meta-regression and subgroup analysis will be conducted to examine the moderating roles of age, gender, culture, health status, and other potential moderators. The characteristics and main findings of eligible studies will be summarized in tables and narrative descriptions. Results from the meta-analysis, meta-regression, and subgroup analysis will be presented quantitatively.

**Results:**

We registered our protocol with PROSPERO, conducted the search, and initiated the screening in April 2024. We expect to start data analysis in October 2024 and finalize the review by March 2025.

**Conclusions:**

The systematic review and meta-analysis will provide evidence on the protective role of self-compassion in resilience under adversity. Our investigation into potential moderators will highlight the contexts and groups where the benefits of self-compassion can be maximized. The findings are expected to provide valuable insights for health care professionals and stakeholders, informing the development of interventions aimed at enhancing resilience by fostering self-compassion.

**Trial Registration:**

PROSPERO CRD42024534390; https://tinyurl.com/3j3rmcja

**International Registered Report Identifier (IRRID):**

PRR1-10.2196/60154

## Introduction

### Background

Individuals in the modern era face a multitude of adversities that can negatively impact their mental well-being, physical health, and social functioning [1-4]. Such adverse life events may contribute to the development of stress-related disorders, including anxiety, depression, and posttraumatic stress disorders [5], and can increase the risk of substance use, eating disorders, and internet addiction [6]. In addition, prolonged activation of the stress response system due to adversity can increase the risk of cardiovascular diseases [7]. However, not all individuals experiencing adversity exhibit negative outcomes. Some individuals possess the capacity to maintain their health or recover swiftly when confronted with adversity, effectively demonstrating resilience in such challenging circumstances. Promoting resilience is vital to prevent stress-related disorders by prioritizing the cultivation of personal strengths [5]. This approach can benefit individuals across different age groups; for instance, it can help prevent children and adolescents from internalizing problems amid interpersonal tensions and academic pressures [8,9], as well as mitigate work-related burnout in adults [10,11]. Therefore, enhancing resilience could be a promising strategy to combat stress-related disorders and facilitate well-being despite pervasive stressors.

Resilience can be conceptualized and operationalized in various ways, with no consensus yet reached among researchers. A total of 3 common approaches are applied to define resilience, including the trait-oriented, process-oriented, and outcome-oriented perspectives [12,13]. Resilience can be defined as a stable trait or ability to recover from adversity; as a dynamic process during which an individual deploys internal and external resources to maintain well-being in adversity; or as adaptive outcomes despite adversity, such as maintained psychological well-being and the absence of psychopathology. Accordingly, as a trait, resilience can be measured using psychometric scales [14]; as a process, it could be modeled as an adaptive trajectory following adversity [15]; and as outcomes, it could be evaluated through measures of mental or physical health accounting for stressor loads [5]. To accommodate various approaches, we define resilient individuals as those possessing the capacity to leverage personal, social, and environmental resources to cope with stressors, thereby facilitating adaptive responses and health outcomes during and after adversity [5,13].

Since the inception of resilience research, considerable attention has been devoted to identifying protective factors that may enhance resilience. One such potential protective factor is self-compassion [16]. Self-compassion refers to treating oneself with warmth and kindness in the face of failures, personal inadequacies, or adversities [17]. According to the self-compassion framework developed by Neff [18], self-compassion contains six components distributed across three dimensions, which are (1) self-kindness versus self-judgment, (2) common humanity versus isolation, and (3) mindfulness versus over-identification. Self-kindness refers to treating oneself with understanding, warmth, and kindness, instead of judging oneself harshly. Common humanity involves recognizing suffering as shared experiences in human beings, rather than perceiving it as isolating and unique to oneself. Mindfulness involves developing an awareness of one’s pain and approaching it with a balanced perspective, thereby avoiding excessive immersion in or over-identification with negative emotions or experiences. Higher levels of self-kindness, common humanity, and mindfulness, and reduced levels of self-judgment, isolation, and over-identification, jointly contribute to a self-compassionate state of mind.

Self-compassion has been proposed to be a potential resilience factor in light of its positive roles in promoting mental health and aiding in the management of stressful situations [19]. Research indicates that self-compassion is positively correlated with psychological well-being and the use of adaptive coping strategies [20,21] while being negatively associated with detrimental symptoms, such as rumination, stress, depression, and anxiety [22]. Furthermore, self-compassion appears to be particularly beneficial during stressful events [23,24]. Evidence suggests that self-compassion could buffer the impact of perceived stress on depression, anxiety, and negative affect up to 6 months later [25], and reduce the negative effect of poor physical health on psychological health [23]. Collectively, these findings indicate that self-compassion could possibly promote resilience under stressful life events.

Preliminary evidence has supported this claim by demonstrating positive correlations between self-compassion and resilience in various populations, including adolescents, college students, and adults [26-28]. Moreover, Gilbert’s [29] affect-regulation model could offer an evolutionary perspective to understand the role of self-compassion as a protective resilience factor. According to this model, self-compassion originates from the safeness system, an evolved mechanism that fosters affiliative affects and a sense of calmness. The safeness system has the capacity to downregulate the threat system, which is hypothesized to activate in potentially threatening situations and elicit arousal responses [29,30]. Gilbert’s [29] theory suggests that self-compassion could mitigate threat-related reactions and induce soothing feelings in stressful situations, thereby promoting resilient adaptation and recovery from adversity.

Potential pathways through which self-compassion is linked to resilience may involve emotion regulation and stress coping [20,31]. First, self-compassion has been shown to reduce negative emotions and enhance positive emotions [21], indicating its potential effectiveness as an emotion regulation strategy. A systematic review identified emotion regulation as a mechanism through which self-compassion contributes to mental health [31]. Second, as suggested in Gilbert’s [29] model, self-compassion may mitigate stress-related reactions, and promote adaptive coping strategies [20,29]. Biological evidence also suggested that self-compassion could reduce stress-related reactions, as indicated by lower levels of salivary alpha-amylase [32], reduced daily cortisol levels, and baseline inflammation [33,34].

The association between self-compassion and resilience may differ across different demographic groups, taking into account characteristics such as age, gender, culture, and health status. Some evidence suggests that self-compassion has a more pronounced effect on well-being as individuals age [35], is more effective in reducing rumination in adult women compared with men [36], and has a greater impact on psychological health in adults with poorer health status [23]. In addition, a cross-cultural study found that self-compassion was a stronger predictor of life satisfaction in Taiwan than in Thailand, indicating a moderating role of cultural backgrounds such as Confucianism and Buddhism [37].

To date, one scoping review has provided an initial overview of the association between self-compassion and resilience in work situations [19]. However, the nonsystematic nature of the search, the practice-oriented focus, and the specific context of this review have limited its ability to comprehensively investigate the evidence. It is also crucial to extend this investigation to other populations, such as emerging adults and the retired population, due to the unique challenges they face [19,38]. Furthermore, despite the existence of meta-analyses assessing the link between self-compassion and well-being or psychopathology [21,39], there is still a lack of quantitative synthesis on the effect size between self-compassion and resilience. In addition, the specific associations between different components of self-compassion and resilience have not been systematically explored. Such an analysis is essential to determine the inclusion of self-compassion in resilience-enhancing interventions and to identify which components of self-compassion should be targeted. Finally, there is limited knowledge about how this relationship may vary across different subgroups, such as those defined by age, gender, culture, and health status. Addressing this gap could help identify which groups are most likely to benefit from self-compassion, thereby informing the design of more targeted and effective interventions.

### Objectives

The primary aim of the systematic review and meta-analysis is to investigate the relationship between self-compassion and resilience. The relationship between 6 components of self-compassion (ie, self-kindness, self-judgment, common humanity, isolation, mindfulness, and over-identification) and resilience, and self-compassion and resilience measured with different methods (eg, measured using scales or as health outcomes related to the stressor), will also be investigated. The secondary aim is to explore the moderating effects of age, gender, culture, and health status in the relationship between self-compassion and resilience, along with other potential moderators. Finally, the potential mechanisms between self-compassion and resilience will be investigated.

## Methods

### Design and Registration

The protocol was developed following the PRISMA-P (Preferred Reporting Systematic Review and Meta-Analysis Protocols) guidelines [40] and the standardized Joanna Briggs Institute approach for conducting systematic reviews of association [41]. The checklist for PRISMA-P can be found in Multimedia Appendix 1. A brief version of this protocol was preregistered in the International Prospective Register of Systematic Reviews (PROSPERO; protocol ID: CRD42024534390).

### Ethical Considerations

This study is a systematic review and meta-analysis based on secondary analysis of publicly available data from previously published studies. No new data collection or direct interaction with human participants will be involved. Therefore, ethical approval will not be required for this study.

### Eligibility Criteria

We developed the inclusion and exclusion criteria following the PEO (Population, Exposure of interest, and Outcome) framework [41], which is applicable in reviews addressing associations between variables. Inclusion and exclusion criteria are summarized in Textbox 1.

**Inclusion and exclusion criteria.**

**Inclusion criteria**

- Study types
  - Peer-reviewed empirical studies using quantitative methods and full texts that explore the association between self-compassion and resilience
- Population
  - General population
- Exposure
  - Self-compassion assessed using validated scales
- Outcome
  - Resilience measured using validated scales, and self-compassion measured as a moderator between a stressor and health outcomes, or other validated measurements
  - Resilience explicitly stated as the study focus or framework

**Exclusion criteria**

- Study types
  - Qualitative studies, conference abstracts, review articles, case reports, and editorials
- Population
  - Nonhuman subjects
- Exposure
  - Studies without a validated measure of self-compassion; studies measuring compassion as a broad concept rather than focusing specifically on self-compassion
- Outcome
  - Studies with no validated measurements of resilience
  - Studies that do not explicitly state resilience as the primary focus or framework

#### Type of Studies

Only peer-reviewed empirical studies using quantitative methods will be included. The study designs will cover observational studies (eg, cross-sectional surveys and longitudinal studies) that investigated the relationship between self-compassion and resilience, and experimental studies involving the manipulation of self-compassion levels. Experimental studies will be synthesized separately to examine the causal relationship between self-compassion and resilience. Qualitative studies, conference abstracts, review articles, case reports, and editorials will be excluded. No restrictions will be applied regarding the language of the studies.

#### Population

We will explore the general population, including individuals of different ages (including children, adolescents, youth, and adults), genders, cultures, and health statuses. Studies involving nonhuman subjects will be excluded.

#### Exposure of Interest

The exposure of interest in this study is self-compassion. The eligible studies should adopt a validated measure of self-compassion, such as the Self-Compassion Scale [18], its short version [42], or youth version [43], and may include measurements of its components (eg, self-kindness, self-judgment, common humanity, isolation, mindfulness, and over-identification). Experimental studies manipulating levels of self-compassion should include manipulation checks using validated scales, such as the State Self-Compassion Scale [44]. Randomized controlled trials (RCTs) will be eligible for inclusion if the interventions are specifically designed to target self-compassion, such as the Mindful Self-Compassion intervention [45]. For studies in which compassion is a key variable, data specifically relating to compassion directed toward oneself, or self-compassion, should be reported. For instance, if the Compassionate Engagement and Action Scales [46,47] were used, the study should include data on the self-compassion subscale. Studies that discuss compassion solely as a broad concept without a focus on self-compassion will be excluded.

#### Outcome

This review will focus on resilience as the primary outcome. We will not limit the definitions or measurements of resilience and will include studies explicitly indicating resilience as a study focus and using validated measurements to assess resilience, such as using resilience scales (eg, the Connor-Davison Resilience Scale [47], the Resilience Scale for Adults [48], Resilience Scale [49], and the Brief Resilience Scale [14]). It is noteworthy that 1 common method to assess resilience is to examine health outcomes related to specific stressful events or stressor loads [12,15]. In this context, the role of self-compassion in promoting resilience may be observed through its moderating effect on the adverse effect of stressful events on health outcomes, even if resilience is not directly measured. We will include studies that use this approach to measure self-compassion as a resilience factor but will exclude those that apply this approach without explicitly focusing on resilience.

### Search Strategy

We will perform a systematic literature search in electronic databases, including Web of Science Core Collection (Clarivate), PsycINFO (Ovid), MEDLINE (Ovid), Scopus, CINAHL (EBSCO), CNKI (Chinese database). No filters will be used during the search stage. Search strings will encompass 2 categories, which are (1) self-compassion, and (2) resilience. For self-compassion, the search terms could be “self-compassion* OR (compassion near/5 (self OR oneself)).” For resilience, the search terms could be “resilient*.” The Boolean operator “AND” will be applied between (1) and (2). The detailed search strategies can be found in Multimedia Appendix 2. Furthermore, we will complement the search by screening similar articles (eg, those recommended by PubMed) and using backward- and forward-chain searching using the top 10 relevant studies to identify any overlooked literature.

### Screening Procedure

Abstract and title screening and full-text screening will be conducted in Covidence, which is a web-based systematic review tool. All search results will be imported into Covidence and deduplicated automatically. The titles and abstracts will be screened independently by 2 reviewers (XL and JH) to evaluate their initial eligibility. The full text of a publication will be retrieved if at least 1 of the reviewers deems it potentially eligible. Following this, 2 reviewers will independently screen the full text according to the eligibility criteria and note down the exclusion reasons. Any disagreement in the screening process will be resolved through discussions between 2 reviewers, and consultation from a senior researcher (GZ). Cohen κ will be calculated to assess the consistency between the 2 reviewers. A flowchart will be drawn to demonstrate the process of screening according to PRISMA [40].

### Data Extraction

A total of 2 reviewers (XL and HX) will independently extract information from the eligible literature in Covidence using a standardized information extraction form. The form will be developed following the PRISMA guideline and revised by piloting on the first few literature. The extracted information will include study characteristics (ie, author and year, title, journal, country, study design, and relevant study aim), population information (ie, demographic information, health status, age range, and mean, and gender proportion), exposure of interest (ie, measurement of self-compassion and its Cronbach α, unadjusted and adjusted effect size, CI and *P* value, and confounders), outcome (measurement of resilience and its Cronbach α, definition, and domain of resilience), main findings, and potential moderators and mediators. In addition, if the literature reported an RCT study, the intervention type, timing, and duration will be extracted. If there are missing data necessary for effect size calculation or quality assessment, the authors will be contacted up to 3 times within a month to request the information.

### Risk of Bias Assessment

The risk of bias of all individual studies and overall evidence will be assessed independently by 2 reviewers (XL and HX). Cohen κ will be calculated to evaluate the interrater reliability. Any discrepancy will be addressed through discussions between 2 reviewers and consultation from a senior reviewer (GZ). Standardized critical appraisal checklists developed by the Joanna Briggs Institute will be applied to evaluate the methodological quality of each study [50]. Different checklists will be adopted based on study design, covering observational study (ie, cross-sectional and longitudinal study), quasi-experimental study, experimental study, or RCT study. The appraisal of observational studies will take into account selection bias, information bias, and confounding, while the appraisal of experimental or quasi-experimental studies will consider selection bias, performance bias, attrition bias, detection bias, and reporting bias [51]. The quality of each study will be rated as high, low, or unclear in each domain. Exclusion will not be implemented based on study quality, but the appraisal outcome will be incorporated in the sensitivity analysis (by omitting studies rated as high risk of bias) to assess the influence of risk of bias on results.

The overall publication bias of evidence in the systematic review will be assessed by drawing a funnel plot and conducting sensitivity analyses using publication bias tests if there are at least 10 studies [52,53]. If the publication bias is identified, the trim-and-fill method will be used to compensate for it [54].

The overall certainty of evidence will be assessed using the GRADE (Grading of Recommendation Assessment, Development and Evaluation) [55]. The GRADE assesses the overall certainty of evidence using 5 indices: risk of bias, inconsistency, indirectness, imprecision, and publication bias [55]. The quality of the evidence will be categorized into 4 degrees: high, moderate, low, or very low.

### Data Synthesis

First, a narrative synthesis will be reported, showing study characteristics, such as year of publication, country, participants and sample size, and study design. Studies with similar study designs and measurement of the outcome (ie, resilience) will be grouped together and summarized in tables. The potential moderators and mechanisms between self-compassion and resilience will be summarized narratively.

A meta-analysis will be conducted on relatively homogeneous studies stratified by study designs and measures (eg, by using scales or calculating moderating effects), if there are at least 5 studies in each analysis [56]. We will assess the heterogeneity of studies in each analysis using *I*^2^ statistic, with a value of larger than 75% indicating a considerable heterogeneity [57]. Due to the broad populations that are eligible for inclusion, heterogeneity is assumed to exist. Therefore, to account for between-study variance, we will apply a random-effect meta-analysis to calculate the pooled effect sizes (ie, correlation coefficients for observational studies, and Cohen *d* for experimental studies) between self-compassion and resilience. The average connection between each component of self-compassion and resilience will also be calculated for studies providing necessary data. The Cronbach α will be used to correct the effect sizes, accounting for measurement unreliability. A forest plot will be drawn to illustrate the confidence interval of the relationship of interest in each study.

Furthermore, subgroup analyses and meta-regression will be conducted to identify potential moderators in this relationship and to address heterogeneity in the evidence. Subgroup analyses will be stratified by age groups (adolescents, young adults, and older adults), gender (women vs men), cultural backgrounds (eg, cultures influenced by Buddhism vs Confucianism; Western vs Eastern cultures), and health status (clinical vs nonclinical populations). In addition, we will compare effect sizes across studies with different study designs and varying methodological quality. A meta-regression will be used to further investigate potential moderators, including age, gender proportion, culture, health status, and risk of bias. As for the potential mediators, if there are multiple studies investigating the same mediator, we will use the meta-analytical structural equation modeling method to synthesize the mediation effect quantitatively [58]. All the statistical analyses will be conducted in R (R Core Team, version 4.2.0), using packages such as metaphor [59] or meta [60].

We will document the search and screening process using the PRISMA flowchart and present the characteristics and main findings of eligible studies through tables and narrative descriptions. The results obtained from meta-analysis, meta-regression, and subgroup analysis will be reported in a quantitative manner.

## Results

We registered our protocol with PROSPERO, conducted the search, and initiated the screening in April 2024. We expect to start data analysis in October 2024 and finalize the review by March 2025.

## Discussion

To the best of our knowledge, this study will be the first systematic review and meta-analysis to comprehensively examine the association between self-compassion (overall and its components) and resilience in the general population. In addition, it aims to explore the moderating influences of age, gender, cultural background, and health status, as well as potential mediators in this relationship. Given the established role of self-compassion in coping with adversity, we anticipate finding a positive association between self-compassion and resilience, along with insights into the relevant moderators and mediators.

Enhancing resilience in the general population is crucial for maintaining psychological and physical well-being amidst diverse stressors and adversities [61,62]. Resilience-focused interventions targeting children and adolescents have been effective in reducing internalizing symptoms, such as anxiety and depressive symptoms [8]. By strengthening their ability to cope with adversity, such as experiences of maltreatment, these interventions can help reduce the intergenerational transmission of depression and provide long-term benefits throughout their lives [63]. Similarly, enhancing resilience in older adults has pronounced effects in promoting successful aging, longevity, and reducing depression [64].

Although there are valuable studies aiming to address the associations between adversity, or risk factors and resilience [65,66], identifying modifiable resilience factors like self-compassion and understanding the underlying mechanisms could offer insights into preventing stress-related disorders in challenging situations before they develop. Considering the conducive role of self-compassion on well-being, coping, and reducing psychopathological symptoms in adversity, it could be a protective factor to promote resilience [16,20].

To date, although there is one scoping review exploring their relationship in work contexts [19] and a chapter providing a narrative overview of this relationship [53,67], a systematic and quantitative examination of this association has not yet been conducted. The absence of such a synthesis could lead to insufficient evidence supporting the targeted use of self-compassion as an intervention for enhancing resilience, as well as an unclear understanding of its potency and the specific roles of its components. Specifically, discerning which component has a stronger connection with resilience and in which group the benefits of self-compassion are more pronounced could inform the design of targeted interventions, maximizing effectiveness and efficiency. The findings of this systematic review and meta-analysis will be applied in practice by disseminating results and insights on intervention design to health care professionals and stakeholders.

## Appendix

Multimedia Appendix 1 PRISMA-P (Preferred Reporting Systematic Review and Meta-Analysis Protocols) checklist.

Multimedia Appendix 2 Search strategies for electronic databases.

## Abbreviations

PEO: Population, Exposure of interest, and Outcome
PRISMA: Preferred Reporting Systematic Review and Meta-Analysis
PRISMA-P: Preferred Reporting Systematic Review and Meta-Analysis Protocols
RCT: randomized controlled trial
GRADE: Grading of Recommendation Assessment, Development and Evaluation

## Data Availability

The datasets generated and analyzed during the systematic review and meta-analysis will be available in the Open Science Framework repository.
