# Supplementary material for: The Relationship Between Self-Compassion and Resilience in the General Population: Protocol for a Systematic Review and Meta-Analysis
Source: JMIR Res Protoc. 2024 Dec 5;13:e60154. doi: 10.2196/60154 (PMC11659685; doi:10.2196/60154)
Supplement: Multimedia Appendix 2 [file resprot_v13i1e60154_app2.docx]

**Search Strategies for Electronic Databases**

**Web of Science Core Collection** (1999-, Clarivate platform)

1: TS=(self-compassion* or (compassion* near/5 self) or (compassion* near/5 oneself))

2: TS=(resilien*)

3: #1 AND #2

**PsycINFO** (1806-, EBSCO platform)

1 self-compassion* or (compassion* N5 self) or (compassion* N5 oneself)

2 resilien*

3 1 and 2

**Medline** (1950-, Clarivate platform)

1: TS=(self-compassion* or (compassion* near/5 self) or (compassion* near/5 oneself))

2: TS=(resilien*)

3: #1 AND #2

**Scopus**

( TITLE-ABS-KEY ( self-compassion* OR ( compassion* W/5 self ) OR ( compassion* W/5 oneself ) ) AND TITLE-ABS-KEY ( resilien* ) )

**CINAHL** (EBSCO platform)

S5 S3 AND S4

S4 S1 OR S2

S3 resilien*

S2 self-compassion* OR (compassion* N5 self) OR ( compassion* N5 oneself )

S1 (MH "Self-Compassion")

**Chinese: CNKI**

(主题: 自我同情 + 自我悲悯 + 自我怜悯 + 自悯 + 自我关怀) AND (主题: 韧性 + 心理韧性)
